# On the Measurement of Climate Change Anxiety: French Validation of the Climate Anxiety Scale

**DOI:** 10.5334/pb.1137

**Published:** 2022-03-22

**Authors:** Camille Mouguiama-Daouda, M. Annelise Blanchard, Charlotte Coussement, Alexandre Heeren

**Affiliations:** 1 Psychological Sciences Research Institute, Université Catholique de Louvain, BE; 2 Le Beau Vallon – Psychiatric Hospital, BE; 3 Institute of Neuroscience, Université Catholique de Louvain, BE

**Keywords:** climate change anxiety, climate change, psychometrics, French validation, eco-anxiety, assessment

## Abstract

The notion of climate change anxiety has gained traction in the last years. Clayton & Karazsia (2020) recently developed the 22-item Climate Change Anxiety Scale (CAS), which assesses climate change anxiety via a four-factor structure. Yet other research has cast doubts on the very structure of the CAS by calling either for a shorter (i.e. 13 items) two-factor structure or for a shorter single-factor structure (i.e. 13 items). So far, these three different models have not yet been compared in one study. Moreover, uncertainty remains regarding the associations between the CAS and other psychological constructs, especially anxiety and depression. This project was designed to overcome these limitations. In a first preregistered study (*n* = 305), we translated the scale into French and tested, via confirmatory factor analyses (CFA), whether the French version would better fit with a four-, two-, or single-factor structure, as implied by previous works. We also examined how the CAS factors related to depression, anxiety, and environmental identity. In a second preregistered study, we aimed at replicating our comparison between the three CFA models in a larger sample (*n* = 905). Both studies pointed to a 13-item version of the scale with a two-factor structure as the best fitting model, with one factor reflecting cognitive and emotional features of climate change anxiety and the other reflecting the related functional impairments. Each factor exhibited a positive association with depression and environmental identity but not with general anxiety. We discuss how this two-factor structure impacts the conceptualization of climate change anxiety.

Given the potential impact of climate change on human well-being and survival (Masson-Delmotte et al., 2018; Steffen et al., 2015), many people have reported experiencing unpleasant emotions and distress about climate change (Taylor, 2020; Van der Linden, 2014; for a review, see Cianconi et al., 2020). Recent research has pointed to climate anxiety (also known as eco-anxiety) as one of the most significant and prevalent emotional responses to the climate crisis (e.g., Clayton & Karazsia, 2020; Stanley et al., 2021).

So far, however, a number of varied definitions have been proposed for delineating climate anxiety. For instance, some scholars have viewed it as an anxiety feeling related to anthropogenic climate change (e.g., Pihkala, 2020) or apprehension and stress about anticipated threats to ecosystems by climate change (e.g., Cunsolo et al., 2020). In contrast, others have construed it as “a chronic fear of a doomed environment” (Clayton et al., 2017) or as “a generalized sense that the ecological foundations of existence are on the brink of collapsing” (Albrecht, 2012, p. 250).

Although recent research has pointed to the inherently multifaceted nature of climate anxiety (e.g., Coffey et al., 2021; Pihkala, 2020), one possible explanation for these various definitions is the intrinsic multidisciplinary nature of the research field on climate change, resulting in inputs arising from various disciplines (e.g., environmental sciences, psychology, philosophy). However, such heterogeneity in defining the construct of climate anxiety is problematic, as it precludes any actual operationalization allowing researchers to compare across studies.

From an empirical psychological perspective, although several measures have been developed to evaluate emotional responses vis-à-vis climate change (e.g., Reser et al., 2012; Searle & Gow, 2010), none focused on climate anxiety per se.^1^ To tackle this issue, Clayton and Karazsia (2020) recently provided the first operationalization of climate anxiety by developing a 22-item English self-report scale for assessing and quantifying climate anxiety, the Climate Anxiety Scale (hereafter CAS).

Clayton and Karazsia (2020) examined the factorial structure of their newly developed scale among US residents via exploratory (*n* = 203 participants) and confirmatory (*n* = 199 participants) factor analyses. Both approaches pointed to a four-factor structure. The first factor (items 1–8) assesses cognitive and emotional difficulties in response to climate change, reflected in rumination, difficulty sleeping or concentrating, crying, or nightmares about climate change. The second factor (items 9–13) taps into the functional impairments and aims to assess whether thinking about climate change has damaged the individual’s ability to socialize, work, or concentrate at work or school. It includes items like “My concerns about climate change interfere with my ability to get work or school assignments done.” The third factor (items 14–16) reflects (direct and indirect) personal experience of climate change. It includes items such as “I have been directly affected by climate change” or “I know someone who has been directly affected by climate change.” Finally, the fourth factor (items 17–22) denotes behavioral engagement and the tendency to deploy adaptive behavioral responses vis-à-vis climate change. It includes items such as “I try to reduce my behaviors that contribute to climate change” or “I feel guilty if I waste energy.” Regarding the metric properties, the internal reliability was high for each subscale (i.e., factor), with Cronbach’s alphas higher than 0.80.

However, uncertainty remains regarding the very structure of the CAS for a few key reasons. First, Clayton and Karazsia (2020) suggested in their article that the first 13 items of their scale—i.e., those of the two first factors—might be more representative of climate anxiety than the four-factor structure. Clayton and Karazsia (2020) explain that they incorporated items related to the experience of climate change and to pro-environmental behaviors to see whether these features were associated with climate anxiety (Clayton & Karazsia, 2020). However, they did not theorize that these features were defining features of climate anxiety nor did they test whether a two-factor structure encompassing the first two factors outperforms their initial four-factor structure. Second, Wullenkord et al. (2021) examined the metric properties of a German short version of the CAS composed of the first 12 items (and not 13 items, since these authors removed item 6).^2^ They found that the two-factor structure suggested by Clayton and Karazsia (2020) yielded less-than-ideal fit indices, although marginally better than a single-factor structure encompassing the first 13 items of the CAS. However, they did not test whether this single-factor structure outperforms the initial four-factor model suggested by Clayton and Karazsia (2020). Altogether, these conflicting findings have led to doubts within the scientific community regarding the very validity of the CAS.

Another key limitation of prior research concerns the concurrent and discriminant validity of the CAS. Clayton and Karazsia (2020) assessed the concurrent and discriminant validity of their scale by examining the patterns of correlations between, on the one hand, each of the four CAS factors and, on the other hand, a general measure of environmental identity (i.e., one’s perception of identification with and emotional connection to nature; Clayton, 2003) as well as a broad mixed measure of depression and anxiety (i.e., a 4-item measure of general anxiety and depression combining them through one sole sum-score). Each factor except for behavioral engagement was positively associated with the mixed measure of depression and anxiety. Moreover, each factor except for functional impairments was positively associated with environmental identity.

However, Clayton and Karazsia (2020) assessed depression and anxiety via a four-item measure combining anxiety and depression through one sole sum-score. Such an approach is problematic, as it precludes any inference regarding the distinct associations between climate anxiety, depression, and anxiety. Moreover, Wullenkord et al. (2021), who used the same four-item measurement tool, dissociated the items of anxiety (2 items) and depression (2 items), found a weak (though significant) correlation between the total score of the German 12-item single-factor scale score and depression (*r* = .21) and anxiety (*r* = .25). However, they only mention this observation in the R code they shared as supplementary materials, and there was curiously no allusion to this observation in their main manuscript. Given the theoretical and clinical relevance of improving our understanding of the potential interplay between, on the one hand, climate anxiety and, on the other hand, general anxiety and depression (e.g., Clayton, 2020), such an absence of consideration for the distinction between anxiety and depression is problematic and deserves a more careful audit.

Finally, none of first three factors of the CAS were associated with the behavioral engagement subscale of the CAS (Clayton & Karazsia, 2020; Wullenkord et al., 2021). This is at odds with previous studies reporting moderate-to-strong associations between climate-anxiety-features and pro-environmental behaviors (e.g., Reser et al., 2012; Verplanken et al., 2020).

The main goal of this project is thus to overcome the limitations of previous research in a twofold fashion. First, we wanted to clarify the factor structure of the CAS in French. To do so, we translated the scale into French and then tested via CFAs whether the French version would better fit the data with a four-, a two-, or a single-factor structure (Clayton & Karazsia, 2020; Wullenkord et al., 2021). To the best of our knowledge, no previous published study has compared these three different models based the CFA in the same study. Moreover, given that French is the official language in 32 countries and territories worldwide, this French translation of the CAS represents a crucial contribution not only for ensuring generalization of the construct validity across samples, languages, and cultures, but also for potential large-scale dissemination and use across the globe. In addition, the last report of the Global Climate Risk Index 2020 (Eckstein et al., 2020), which gauges to what extent countries and regions are impacted by climate-related change (e.g., severe storms, floods, heatwaves, droughts), ranked several countries with French-speaking territories among the top 10 most affected places by climate change (e.g., Canada, Madagascar, Rwanda). It thus highlights the urgency of developing French-speaking tools for the assessment of mental health issues in the context of the climate crisis.

Secondly, we wanted to examine whether the French version of the CAS would exhibit a pattern of correlations with depression, anxiety, and environmental identity in line with the initial English version of the scale. Following Clayton and Karazsia (2020), we predicted that each subscale except for behavioral engagement would positively correlate with depression and anxiety. We also predicted that each subscale except for functional impairments would be positively associated with the environmental identity. However, in contrast to Clayton and Karazsia (2020), we decided not to assess depression and anxiety together. Instead, we considered depression and anxiety separately and assessed them using well-validated tools. By doing so, we aimed to gauge the respective associations of depression and anxiety with each factor of the CAS.

## Translation of the scale into French

We followed the guidelines for test adaptation detailed by Hambleton and colleagues (2004). We first translated the items into French and then back-translated them into English. Three fully bilingual experts translated the original English scale into French using a committee approach. The French version was then translated back into English and reevaluated by another bilingual expert. The first author supervised the entire translation/backtranslation process. We asked another expert to verify the conformity of the retranslated English version with the original version and the precision of the French items. Items with problematic back-translation were thoroughly discussed and appropriately amended. Most discrepancies were minor, involving the choice between two synonyms. The French version of the scale is available via the Open Science Framework (OSF) at *https://osf.io/vj9ta/*.

## Study 1

Note that we had one important deviation from our preregistration. We initially planned to focus on testing only the four-factor structure of the CAS in a French-speaking sample. However, when analyzing our data, we discovered the study of Wullenkord et al. (2021) and decided to oppose the four-factor structure with a two- and a single-factor structure. However, because these analyses were not preregistered and were therefore conducted in a post-hoc fashion, we decided to run a second study (Study 2; see below) wherein we preregistered all hypotheses and analyses in an *a priori* fashion (*https://osf.io/uq8gh*). This second study allowed us to examine the replicability and integrity of these three structural models in a second independent larger French-speaking sample.

### Open science practices

The study design, data collection, and analysis plan were preregistered on the Open Science Framework (OSF) at *https://osf.io/5pnvu*. The anonymized data and the R code used for analyses are available on the OSF at *https://osf.io/m3ygz/*.

### Method

#### Sample Size

In their initial scale validation, Clayton and Karazsia (2020) relied on a sample of 200 participants. However, in keeping with scale adaptation and validation guidelines, we opted to follow Rouquette and Fallissard (2011) and not rely on a sample size lower than 300 participants. As a result, our a priori targeted sample size was at least 300 participants (as specified in our preregistration).

#### Participants

We recruited 390 French-speaking participants from the general community via online social media and listserv advertisements. However, before data analysis, we excluded 85 participants with missing values, resulting in a final sample of 305 participants (72.13% women, 26.89% men, and 0.98% others). Participants were between the age of 17 and 70 (*M* = 30.80, *SD* = 11.32). Regarding nationality, 89.18% (*n* = 269) were from France, 6.89% (*n* = 21) from Belgium, .98% (*n* = 3) from Switzerland, .33% (*n* = 1) from Gabon, and 2.62% (*n* = 8) form other French-speaking countries and territories. Their years of education completed since primary school ranged from 3 to 28 (*M* = 16.05, *SD* = 3.08).

#### Procedure

Participants first completed questions regarding their age, gender, nationality, and years of education. Then, they completed the French version of the CAS (see above), the Environmental Identity scale (EIDS; Clayton, 2003), the Generalized Anxiety Disorder-7 (GAD-7; Spitzer et al., 2006), and the Beck Depression Inventory-II (BDI-II; Beck et al., 1996). The study was approved by the Institutional Review Board (Reference: Project IPSY 2021–12) and conducted according to the Declaration of Helsinki. Each participant provided written informed consent before completing the survey.

#### Measures

**GAD-7.** This is a widely used 7-item scale for assessing generalized anxiety symptoms over a two-week period (Spitzer et al., 2006). Participants rate each item (e.g., trouble relaxing, worrying too much) on a 4-point Likert-type scale, from 0 (*Never*) to 3 (*Almost every day*). For each item, a higher score reflects a greater endorsement of the anxiety symptom covered by the item. We used the validated French self-report version of the scale (Micoulaud-Franchi et al., 2016). The internal reliability of GAD-7 was high in the present sample, with a Cronbach’s alpha of .92.

**BDI-II.** This is a 21-item instrument designed to measure both the presence and severity of depressive symptoms over a two-week period (Beck et al., 1996). Each item consists of a group of four statements measuring the symptoms of depression (e.g., loss of interest; sleep problems; self-dislike) that range in intensity, with each item scored on a scale value of 0–3. We used the validated French version of this scale (BDI-II; Beck et al., 1998). The internal reliability of BDI was high in the present sample, with a Cronbach’s alpha of .92.

**EIDS.** This is an 11-item scale assessing environmental identity (Clayton, 2003). Participants rate each item (e.g., I feel that I have a lot in common with other species; I think of myself as a part of nature, not separate from it) on a 7-point Likert-type scale, from 0 (*Not all true for me*) to 6 (*Totally true for me*). We used the validated French version of the scale (Prévot et al., 2018). The internal reliability of the EIDS was high in the present sample, with a Cronbach’s alpha of .84.

#### Data Analysis Strategy

**Normality check.** Only one item of the CAS (i.e., item 7 “I write down my thoughts about climate change and analyze them”) violated normality according to benchmarks of skewness between –2 to + 2 and kurtosis between –7 to +7 (Curran et al., 1996). We accordingly implemented the Satorra-Bentler adjustment procedure in the c^2^ estimation procedure (see below). The skewness and kurtosis of each item are provided in Table S1 in the Supplementary Materials section.

**Confirmatory factor analyses.** We first ran a confirmatory factor analysis (CFA) to investigate whether the French version would better fit with a four-, a two-, or a single-factor structure, as implied by previous research on the CAS (Clayton & Karazsia, 2020; Wullenkord et al., 2021). We did so using the R-package *lavaan* (Rosseel, 2012).

Following Kline (2005), the goodness of fit was not only tested via c^2^ test (implemented with the Satorra-Bentler adjustment^3^ to account for multivariate non-normality; Finney & DiStefano, 2013), but also through the Standardized Root Mean Square Residual (SRMR), the Root Mean Square Error of Approximation (RMSEA), the Comparative Fit Index (CFI), and the Tucker Lewis index (TLI).

SRMR and RMSEA are both residuals-based absolute fit measures. As argued by Hu and Bentler (1998), the combination of RMSEA and SRMR is helpful because the SRMR is sensitive to the misspecification of factor covariances whereas the RMSEA is sensitive to the misspecification of factor loadings. Thus, if both indices are acceptable, then the latent and the measurement models would be considered well specified. Furthermore, the RMSEA has the advantage of typically being associated with a confidence interval. Browne and Cudeck (1992) suggested that RMSEA values close to 0 represent an optimal fit, while RMSEA values equal to or below .05 represent a good fit, RMSEA values between .05 and .08 an adequate fit, RMSEA values between .08 and .10 a mediocre fit, and RMSEA values higher than .10 a non-acceptable fit.

SRMR values are expected to stay below .05 (Kline, 2005). The CFI is an incremental relative fit measure, and values between .95 and 1.0 indicate a good model fit, whereas values ranging between .90 and .95 denote acceptable fit (Bentler, 1990; Hu & Bentler, 1999). Finally, the TLI (also known as the non-normed fit index) represents the discrepancy between the c^2^ value of the hypothesized model and the c^2^ value of the null model (Bentler, 1990). TLI values range between 0 and 1, with a value of .90 or greater indicating a good model fit (Hu & Bentler, 1999).

We also reported the Akaike Information Criterion (AIC; Akaike, 1987) and the Expected Cross-Validation Index (ECVI; Browne & Cudeck, 1989), which are the most suited for comparing non-nested models (Blunch, 2008). AIC and ECVI are fit measures based on information theory. These indices are not used for judging the fit of a single model but are used in situations where there are several realistic but different models from which to choose. These indices are a function of both model complexity and goodness of fit: low scores refer to simple, well-fitting models, whereas high scores refer to complex, poor-fitting models (e.g., Lannoy et al., 2014). Therefore, in a comparison-model approach, the model with the lower score is to be preferred. All the CFAs and related analyses were performed using the R package *lavaan* (Rosseel, 2012).

**Internal reliability.** We computed both the Cronbach’s alpha and McDonald’s omega^4^ coefficients for the global scale and each possible subscale. For both indices, a coefficient with a value higher than .75 reflects good internal reliability (Nunnally, 1978). We also examined the respective impact of each item’s removal on internal reliability. When removing an item, the increase of the total scale (or subscale)’s internal reliability indicates that this item might be potentially problematic in term of internal consistency. We performed these analyses using the R package *psych* (Revelle, 2012).

**Convergent and divergent validity.** We computed Pearson product-moment correlations between each pair of variables of interest. We applied the Benjamini–Hochberg correction (Benjamini & Hochberg, 1995) to hold the false discovery rate (i.e., the expected proportion of falsely rejected null hypotheses) at 5% for the twelve correlations estimated. All of these analyses were performed using R built-in functions.

### Results

The mean, standard deviation, range, skewness, and kurtosis of each item is available in Table S1 in the Supplementary Materials sections.

#### Comparison of the three factor-structures

We examined the three structural models, as implied by previous studies (Clayton & Karazsia, 2020; Wullenkord et al., 2021): a four-factor model of the entire scale (hereafter, 4-factor model); a two-factor structure of the first 13 items (hereafter, 2-factor model); and a single-factor structure of the first 13 items (hereafter, 1-factor model). ***Table 1*** displays the fit indices of the three models.^5^

**Table 1:** Comparison of three CFA Models (Study 1).


MODEL	SATORRA- BENTLER χ^2^	DF	SRMR	RMSEA	RMSEA 90% CI	CFI	TLI	AIC	ECVI

4-Factor	428.53 **	203	.07	.06	.057–.074	.88	.87	16184.17	1.97

**2-Factor**	149.04**	**64**	**.05**	**.07**	**.061–.093**	**.92**	**.91**	**9134.79**	**0.84**

1-Factor	184.40**	65	.05	.09	.075–.106	.89	.87	9178.17	0.98


*Note*: 4-Factor = four-factor model of the 22-item scale; 2-Factor = two-factor model of the 13-item scale; 1-Factor = single-factor model of the 13-item scale. df = degree of freedom; CI = confidence interval; SRMR = Standardized Root Mean Square Residual; RMSEA = Root Mean Square Error of Approximation; CFI = Comparative Fit Index; TLI = Tucker Lewis Index; AIC = Akaike Information Criterion; ECVI = Expected Cross-Validation Index. The best fitting model is shown in bold. **p < .01.

Although the 4-factor and the 1-factor models showed partially acceptable fit indices, the 2-factor model yielded satisfactory ones. Moreover, the 2-factor model exhibited better fit indices that the two other models. Finally, the AIC and ECVI were favorable toward the 2-factor model.

The factor loadings of the three models are, respectively, available in Table S2, Table S3, and Table S4 in the Supplementary Materials sections. The standardized factor loadings of the 2-factor model were all statistically significant (*p* < .001). However, one item (i.e., item 7: “I write down my thoughts about climate change and analyze them”) showed loadings below .40. Therefore, we also reran all analyses without this item. However, results did not show any substantial change in fit index values (see Table S5 in the Supplementary Materials sections). To be consistent with the initial scale, we thus did not exclude item 7.

### Internal Reliability

With Cronbach’s alpha and McDonald omega coefficients higher than 0.75, the internal reliability was high for each of the two factors of the 2-factor model. The Cronbach’s alphas were .84 for the cognitive emotional factor and .82 for the functional factor (.90 for the first 13 items as a whole), and the McDonald omegas were .89 for the cognitive emotional factor and .85 for the functional factor (.91 for the first 13 items as a whole). For each factor (and the 13-item scale as a whole), Cronbach’s alpha coefficients decreased if any of the items were deleted.

For transparency and ease of comparison with other works, we also examined the internal reliability of the two other factors, as implied by the four-factor model preferred by Clayton and Karazsia (2020). The Cronbach’s alphas for the third factor (“Experience of climate change”) and the fourth factor (“Behavioral engagement”) were .80 and .63, respectively, while their respective McDonald omega coefficients were .82 and .80. The Cronbach’s alpha for the 22-item version of the scale (as a whole scale) was .86, while the McDonald omega coefficient was .89.

### Correlations Between the CAS and Other Constructs

***Table 2*** shows the correlations between the two subscales of 2-factor model, the GAD, the BDI, and the EID. Cognitive-emotional impairments and functional impairments were positively associated with depression and environmental identity. In contrast to our predictions, neither the cognitive-emotional factor nor the functional factor was associated with general anxiety.

**Table 2:** Correlations between the Climate Change Anxiety (sub)scale and other psychological constructs (values reported between brackets denote the 95% confidence intervals of the correlations).


	CEI	FI	BDI	GAD	EID

CAS (13-item)	.95**[.94–.96]	89**[.87–.91]	30**[.18–.39]	.02[–.09–.12]	.34**[.23–.43]

CEI (Factor 1)		.73**[.68–.78]	.28**[.17–.38]	.05[–.05–.16]	.34**[.23–.43]

FI (Factor)	.73**[.68–.78]		.27**[.16–.37]	–.03[–.15.–07]	.29**[.18–.39]

EX (Factor 3)	.43**[.33–.52]	.37**[.27–.46]	.18[.07–.29]	.03 [–.07–.14]	.33**[.00–.22]

BE (Factor 4)	.43**[.34–.52]	.41**[.31–.50]	.10 [-.00–.21]	.11 [.00–.22]	.29**[.18–.39]


*Note*: CEI = cognitive-emotional impairments; FI = Functional impairments; EX = Personal experience of climate change; BE = Behavioral Engagement; BDI = Beck depression inventory. GAD = general anxiety disorders; EID = environmental identity; CAS = climate anxiety scale. * *p* < .05 (corrected for multiple comparisons using the Benjamini-Hochberg procedure).

### Discussion of Study 1

The main goal of this study was to clarify the factor structure of CAS in a French-speaking sample. Our findings pointed to a two-factor structure encompassing the first 13 items as the best model compared to a four-factor model (encompassing the full scale) and to a single-factor model (based on the first 13 items). However, although the 2-factor model exhibited the best fit indices values, those of the 1-factor model were only slightly inferior to those of the 2-factor model. As such, uncertainty remained regarding the structure of the CAS, and we decided to re-run these three CFA models and compare them in a second independent study, relying on a larger sample.

## Study 2

This study aims to replicate the investigation into the three different factor structures investigated in Study 1. This study was pre-registered on OSF at *https://osf.io/uq8gh*. The anonymized data and our R code used for analyses are also available on OSF, at *https://osf.io/m3ygz/*.

### Method

#### Participants

We recruited 912 French-speaking participants using a procedure similar to Study 1. We excluded 7 participants with missing values before data analysis (that is, participants who did not complete the entire survey), resulting in a final sample of 905 participants (55.25% women, 44.31% men, and .44% others). Participants were between 17 and 77 years old (*M* = 36.89, *SD* = 11.87). Regarding nationality, 91.60% (*n* = 828) were from France, 4.31 % (*n* = 39) were from Belgium and 4.09 % (*n* = 37) form other countries. The years of education completed since primary school ranged from 4 and 65 (*M* = 16.68 *SD* = 3.34).

#### Measures and Procedure

Participants first completed a few questions regarding their age, gender, nationality, and years of education. Then, they completed the French version of the CAS (see above). The study was approved by the Institutional Review Board (reference: Project IPSY 2021–12) and conducted according to the Declaration of Helsinki. Each participant provided written informed consent before completing the survey.

#### Data Analysis Strategy

**Normality check.** None of the item of the CAS violated normality according to benchmarks of skewness between –2 to + 2 and kurtosis between –7 to +7 (Curran et al., 1996). However, to align with Study 1, we implemented the Satorra-Bentler adjustment procedure in the c^2^ estimation procedure (see Study 1). The skewness and kurtosis of each item is available in Table S7 in the Supplementary Materials.

**CFA models.** As in Study 1, we tested three structural models: a four-factor model of the entire scale (hereafter, 4-factor model), a two-factor model of the first 13 items of the CAS (hereafter, 2-factor model), and a single-factor model of the first 13 items (hereafter, 1-factor model). The fit indices were computed as in Study 1. We also examined the internal reliability of the best fitting model as in Study 1.

### Results

The mean, standard deviation, range, skewness, and kurtosis of each item are available in Table S7 of the Supplementary Materials.

#### Comparison of the three factor-structures

***Table 3*** displays the fit indices of the three models. As in Study 1, although the 4-factor and the 1-factor models showed partially acceptable fit indices, the 2-factor model yielded very satisfactory ones and exhibited better fit indices than the two other models.^6^

**Table 3:** Comparison of three CFA Models (Study 2).


MODEL	SATORRA-BENTLER χ^2^	DF	SRMR	RMSEA	RMSEA 90% CI	CFI	TLI	AIC	ECVI

4-Factor	962.74**	203	.07	.06	.063–.072	.86	.84	52084.20	1.28

**2-Factor**	390.48**	**64**	**.05**	**.08**	**.076–.092**	**.89**	**.87**	**31170.33**	**0.60**

1-Factor	542.26**	65	.06	.10	.093–.109	.84	.81	31355.91	0.80


*Note*: df = degrees of freedom; CI = confidence interval; SRMR = Standardized Root Mean Square Residual; RMSEA = Root Mean Square Error of Approximation; CFI = Comparative Fit Index; TLI = Tucker Lewis Index; AIC = Akaike Information Criterion; ECVI = Expected Cross-Validation Index. The best fitting model is shown in bold. ** *p* < .01.

The standardized factor loadings of the 2-factor model were all statistically significant (*p* < .001). The factor loadings of the 4-factor, 2-factor, and 1-factor models are, respectively, presented in Table S8, Table S9, and Table S10 of the Supplementary Materials sections. As in Study 1, with respect to the 2-factor model, item 7 had a factor loading below .40. However, rerunning all analyses without this item did not substantially change the fit index values (see Table S11 in the Supplementary Materials sections). As in Study 1, we decided to not exclude this item to be consistent with the initial scale of Clayton and Karazsia (2020).

#### Internal Reliability

The internal reliability of each subscale was likewise high, with Cronbach’s alphas of .79 and .81 for the cognitive-emotional factor and the functional factor, respectively (and with McDonald Omega coefficients of .83 and .84, respectively). For each factor (and the 13-item scale as a whole), the coefficients decreased if any of the items were deleted. Of note, with a Cronbach’s alpha coefficient of .87 and a McDonald Omega coefficient of .89, the internal reliability of the 13-item total scale score was likewise high. The Cronbach’s alpha for the 22-item version of the scale was .86, and the McDonald Omega coefficient was .89.

#### Correlations between the CAS subscales

We found that the cognitive-emotional impairments (Factor 1) and functional impairments (Factor 2) had a moderate-to-strong correlation, r(903) = .66, p < .001 (corrected for multiple comparisons using the Benjamini-Hochberg procedure). Both “experience of climate change” and “behavioral engagement” subscales showed small positive correlations (all rs < .40, p < .001 corrected for multiple comparisons using the Benjamini-Hochberg procedure) with the cognitive-emotional and functional impairments subscales. All correlations are provided in Table S13 in the supplementary materials).

## General Discussion

We had two primary goals in this project. First, we aimed to clarify the factor structure of the CAS in a French-speaking community sample. To the best of our knowledge, this is the first publication of a French version of the CAS. Moreover, to the best of our knowledge, no previously published study has relied on CFAs to compare the different structural models in the same study. A second goal was to clarify the relations of CAS factors with depression, anxiety, and environmental identity.

Regarding the factor structure of the CAS, we investigated whether our data, via two independent studies, would better fit a four-, two-, or single-factor structure model, as implied by prior studies (i.e., Clayton & Karazsia, 2020; Wullenkord et al., 2021). In our two studies, we consistently found that a two-factor structure, including the 13 first items of the CAS and reflecting the cognitive and emotional features of climate change anxiety (Factor 1) and functional impairments resulting from climate change (Factor 2), best fit our data. In addition, this two-factor model outperformed both the initial four-factor model (with 22 items) and the single-factor structure (with the 13 items denoting climate anxiety viewed as a single latent entity). However, despite these statistical observations, it should be noted that the terms used by Clayton & Karazsia (2020) to label these two factors do not entirely align with contemporary research on anxiety (for a review, see Norton & Paulus, 2017), wherein such a distinction between the cognitive-emotional and functional impairments is unusual. As such, a critical next step in future iterations would thus be to further establish the theoretical and clinical relevance of distinguishing these two features of climate anxiety. Moreover, since psychological mechanisms fluctuates over time (e.g., Blanchard et al., 2022; Heeren et al., 2015), it makes sense to also look at their temporal variations. In the same vein, one may wonder about their temporal unfolding. For instance, does the onset of functional impairments require the precedence of cognitive and emotional impairments? Future research avenues will hopeful attempt to answer these questions.

The second goal of this study was to clarify the relations of CAS factors with environmental identity, depression, and anxiety. Concerning environmental identity (i.e., one’s perception of identification with and emotional connection to nature, Clayton, 2003), we found that it positively correlates with both factors. This is not surprising, since people who feel connected to nature are likely to be more attentive and impacted by threats to their environments (for a discussion, see Clayton, 2003). Our observation is also in line with prior research pointing to environmental identity as an incremental process of climate change anxiety, including functional impairments (Dean et al., 2018). However, our findings are at odds with Clayton and Karazsia (2020), who found that environmental identity was only associated with the cognitive-emotional features (Factor 1) but not with functional impairments (Factor 2). One may wonder about the cultural differences between Clayton and Karazsia’s (2020) samples and ours as a potential explanation. Indeed, the former were US residents, whereas ours are French speakers from different countries but primarily European ones. A critical next step would thus be to clarify whether similar results appear in other languages, cultural backgrounds, and geographic areas.

Moreover, both the cognitive-emotional features (Factor 1) and the functional impairments (Factor 2) correlated with the experience of climate change and pro-environmental behaviors. This observation of a positive correlation between these two factors and pro-environmental behaviors is at odds with Clayton and Karazsia (2020), who did not find such an association. Note that this discrepancy is not surprising and may reflect the current existence of two contrasting views of the relations between climate anxiety and pro-environmental behaviors in the literature. On the one hand, some scholars envision climate anxiety as a potentially adaptive feeling that can foster people’s engagement in pro-environmental behaviors, while on the other hand, others view it as a potentially maladaptive feeling that can inhibit people from engaging pro-environmentally (for discussion, see Doherty & Clayton, 2011; Heeren et al., 2021; Verplanken et al., 2020).

Concerning depression and anxiety, we considered them separately to assess their respective associations with each factor. We found that both factors exhibited positive correlations with depression but not with anxiety. Because Clayton and Karazsia (2020) did not dissociate depression and anxiety, one may thus not exclude that climate change anxiety is, above all, associated with depression and not with anxiety. Although our observations require additional confirmation from future studies with larger and more representative samples, such a possibility aligns with other research suggesting that depressed feelings might play a role in how people experience worries about climate change. For instance, a recent study conducted in Norway (e.g., Marczak et al., 2021) revealed that people who self-identified as worried about climate change exhibited emotional patterns tainted by features more typically associated with depression than anxiety, such as lowered mood, pessimism, loneliness, hopelessness, and guilty feelings. On the other hand, the 13 first items of CAS were loosely based on the Ruminative Response Scale (Treynor, Gonzalez, & Nolen-Hoeksema, 2003) and the functional impairment scale (Weiss, 2000), whose correlations with depression are relatively high, regardless of the climate change context (e.g., Parola et al., 2017). One may thus wonder whether the correlations between the CAS factors and depression do not merely reflect the sources of the CAS items development (see also Wullenkord et al. 2021 for a similar discussion).

Moreover, regarding the absence of association between the first two factors of the CAS and anxiety, it is worth noting that this latter was assessed using the GAD-7 (Spitzer et al., 2006). Although it is one of the most commonly used tools in epidemiological anxiety research (e.g., Schalet et al., 2014), it focuses on generalized anxiety disorder symptoms and does not cover all anxiety-related phenomena. One may thus wonder whether climate change anxiety might not be better associated with other anxiety-related phenomena, like trait anxiety. On the other hand, the GAD-7 covers features of anxiety that are considered transdiagnostic to anxiety and stress-related disorders (e.g., excessive worries, trouble relaxing; Coussement & Heeren, 2022; Norton & Paulus, 2017). Additional research is thus required to further establish whether climate change anxiety is more associated with depression- than anxiety-related phenomena.

The present study has limitations. First, although the current study included participants from different French-speaking countries and territories worldwide, most came from European French-speaking countries. Because French-speakers living in non-European countries and territories might be exposed to more direct impacts of climate change in their environment (e.g., French-speaking African countries), they might vary in emotional response to climate change. A critical next step would thus be to examine the structural invariance of the CAS across these groups. Moreover, since the very experience of climate change anxiety may differ widely across cultural backgrounds (for a discussion, see Coffey et al., 2021; Ojala et al., 2021), it might also be relevant not only to translate the scale but to culturally adapt the CAS to the specificity of each cultural group.

In the same vein, a second limitation is that our study was underpowered (and not designed; see our preregistration) to examine whether the correlations between the CAS scores (and subscores) and environmental identity vary as a function of the cultural background of the respondents. This is unfortunate, since several studies reported that people directly affected by climate change or who feel more connected to the natural world for cultural (e.g., Indigenous communities; Cunsolo Willox et al., 2012) or personal reasons (e.g., farmers; Carleton, 2017) show more intense and prolonged emotional responses to climate change. As such, another critical step would be to examine the potential mediating role of environmental identity in the link between the experience of climate change and climate change anxiety, as well as to assess whether these correlations differ between those who feel more connected to nature versus those who do not.

Third, we did not examine whether the factor structure of the CAS varies based on gender. However, prior research has suggested that women might be more vulnerable to climate change impact (World Health Organization, 2014; Intergovernmental Panel on Climate Change, 2014), likely due to their perceived relative lack of power when facing a threat in many cultures (World Health Organization, 2014). Moreover, gender difference can also be seen through women’s negligible participation, in many countries, in decision-making structures and limited access to and control of agricultural lands, inputs, and services as resources that would foster their adaptation to climate change (World Health Organization, 2014). However, gender differences in climate anxiety have seldom been investigated. To the best of our knowledge, Clayton and Karazsia (2020) were the only ones to examine this question. Through two US samples, they reported no gender differences in climate anxiety. A critical next step would thus be to test, through large-sample studies, whether the CAS structure varies across genders.

Fourth, we relied on self-report measures only. Future studies should examine the correlation between the CAS factors and behavioral and psychophysiological (e.g., skin conductance, cortisol release) responses when exposed to climate-change-related stimuli (for a review on the psychophysiological assessment of stress and anxiety-related phenomena, see Tolin et al., 2021). Likewise, we adapted the CAS, while other scales assessing climate anxiety have been published since we initiated this project. For instance, Hogg et al. (2021) recently developed a 13-item scale that encompasses four factors (i.e., affective symptoms, rumination, behavioral symptoms, and anxiety about one’s negative impact on the planet). A critical next step would thus be to examine whether those different measurement tools tap onto the same construct (for an example of such a methodological approach in another domain, see Desmedt et al., 2021).

Finally, although the notion of climate anxiety (also known as eco-anxiety) has been gaining traction in the media and the scientific literature, uncertainty remains regarding the very nature of this construct (for a discussion, see Coffey et al., 2021). In a recent scoping review of the literature on the notion of eco-anxiety, Coffey et al. (2021) identified more than ten distinct operationalizations of this notion in the existing literature, with most of the literature coming from groups of researchers located in Western countries, thus suggesting a striking lack of consensus between authors regarding the notion of eco-anxiety. Here, we aligned with Clayton and Karazsia (2020)’s operationalization that focuses on anxious feelings associated with perceptions about climate change, even among people who have not personally experienced any direct impacts. However, other operationalizations have been proposed, and one may wonder whether the distinction between the cognitive-emotional features and the functional impact would remain across the plethora of different operationalizations of eco/climate anxiety. On the other hand, this issue also stresses the paucity of theoretical developments and the lack of integrative models regarding climate anxiety. As in any field of science, the absence of clearly testable and falsifiable theories hinders scientific advancement. A vital effort for further progress in this field would thus be to rely on theory construction tools to delineate the fuzzy borders between the many notions related to eco- and climate anxiety available in the existing literature. This would then allow researchers to forge a set of theoretical principles that can be putatively confirmed or rejected via hypothesis-driven research (for details regarding such a methodology, see Borsboom et al., 2021).

These limitations notwithstanding, we provided the first validation of the CAS in a French-speaking community sample. We found that a two-factor structure including only the first 13 items best fit our data.

## Data Accessibility Statement

The study design, hypotheses, data collection, and analysis plan of the two studies depicted in this paper were preregistered on the Open Science Framework (Study 1: *https://osf.io/5pnvu*; Study 2: *https://osf.io/uq8gh*). The anonymized data, as well as the R code used for analyses, are also available on the Open Science Framework (*https://osf.io/m3ygz/*).

## Additional File

The additonal file for this article can be found as follows:

10.5334/pb.1137.s1 Supplemental Tables. Tables S1–S13.

## Footnotes

But see Hogg et al. (2021), who published a new 13-item scale tapping onto climate anxiety while we were revising the manuscript version of this paper.

In an exploratory fashion, they found that item 6 (“I go away by myself and think about why I feel this way about climate change”) had both a very low factor loading and low commonality in the German version and recommended dropping it—a recommendation associated with improved fit indices in their sample (Wullenkord et al., 2021).

Note that we did not plan to implement this adjustment in our preregistration. However, because this adjustment yields more robust findings in case of normality violations, we decided to implement this adjustment. We are thankful to an anonymous reviewer for this thoughtful suggestion.

Note that we did not initially plan to compute the McDonald omega coefficient (see our preregistration). However, Cronbach’s alpha depends on the assumptions that all (unstandardized) loadings are the same (tau-equivalent) and that all error variances of the items are uncorrelated (e.g., Zinbarg et al., 2006). Although the Cronbach’s alpha coefficient is largely used in psychological research, its underlying assumptions are not realistic in many cases, and it may therefore result in an underestimation of the actual internal reliability. We therefore decided to additionally report the McDonald omega coefficient, which does not require tau-equivalence or uncorrelated error variances. We are thankful to an anonymous reviewer for this suggestion.

We also checked for potential univariate outliers. To do so, we first identified participants with values on the first 13 items below or above 2.5 standard deviations from the mean. We identified 7 participants with outlier values. We then re-ran the analyses without those participants, and their exclusion did not lead to any substantial differences in the pattern of findings observed. To be consistent with our preregistration and align with Clayton and Karazsia (2020), we reported the models without excluding those participants. However, the fit indices of the models estimated without the participants with outlier values are available in the supplementary materials (see Table S6).

As in Study 1, we checked for potential univariate outliers. To do so, we first identified participants with values on the first 13 items below or above 2.5 standard deviations from the mean. We identified 7 participants with outlier values. We then re-ran the analyses without those participants, and their exclusion did not lead to any substantial differences in the pattern of findings observed. We therefore report the models without excluding participants, so as to be consistent with our preregistration and align with Clayton and Karazsia (2020). However, the fit indices of the models estimated without the participants with outlier values are available in the supplementary materials (see Table S12).
