# Supplementary material for: On the Measurement of Climate Change Anxiety: French Validation of the Climate Anxiety Scale
Source: Psychol Belg. 2022 Mar 22;62(1):123–35. doi: 10.5334/pb.1137 (PMC8954884; doi:10.5334/pb.1137)
Supplement: Supplemental Tables. — Tables S1–S13. [file pb-62-1-1137-s1.pdf]

SUPPLEMENTARY MATERIALS

On the Measurement of Climate Change Anxiety: French Validation of the Climate Anxiety Scale

Camille Mouguiama-Daouda[1], M. Annelise Blanchard[1], Charlotte Coussement[1,2], & Alexandre Heeren[1,3]

[1] Psychological Sciences Research Institute, Université Catholique de Louvain, Belgium

[2] Le Beau Vallon – Psychiatric Hospital, Belgium

[3] Institute of Neuroscience, Université Catholique de Louvain, Belgium

***Table S1.* Descriptive Statistics for each item (Study 1)**

Items	Mean	SD	Min	Max	Range	Skewness	Kurtosis
CAS1	2.26	1.03	1	5	4	0.43	-0.54
CAS2	2.08	1.07	1	5	4	0.80	-0.14
CAS3	1.57	0.88	1	5	4	1.50	1.56
CAS4	1.50	0.81	1	4	3	1.42	0.94
CAS5	2.20	1.16	1	5	4	0.50	-0.93
CAS6	1.62	0.90	1	4	3	1.22	0.32
CAS7	1.36	0.77	1	5	4	2.25	4.80
CAS8	1.78	1.00	1	5	4	1.15	0.57
CAS9	1.59	0.88	1	5	4	1.44	1.41
CAS10	2.31	1.18	1	5	4	0.33	-1.11
CAS11	1.41	0.71	1	5	4	1.87	3.55
CAS12	1.55	0.83	1	5	4	1.51	1.67
CAS13	1.53	0.91	1	5	4	1.70	1.99
CAS14	1.78	1.09	1	5	4	1.22	0.44
CAS15	1.83	1.18	1	5	4	1.16	0.08
CAS16	2.36	1.24	1	5	4	0.35	-1.12
CAS17	3.57	1.07	1	5	4	-0.57	-0.25
CAS18	4.43	0.89	1	5	4	-1.68	2.54
CAS19	4.64	0.59	2	5	3	-1.60	2.45
CAS20	4.16	0.75	1	5	4	-0.63	0.32
CAS21	3.84	1.08	1	5	4	-0.79	0.01
CAS22	3.56	1.13	1	5	4	-0.28	-0.87

Table S2. **Factor Loadings for the 4-Factor Model (Study1)**

Items	CEI Factor	FI Factor	EX Factor	BE Factor
Il m'est difficile de me concentrer lorsque je pense au changement climatique (item 1)	.69			
Il m'est difficile de m'endormir lorsque je pense au changement climatique (item2).	.75			
Je fais des cauchemars à propos du changement climatique (item 3).	.69			
Je me retrouve à pleurer à cause du changement climatique (item 4).	.69			
Je me demande pourquoi est-ce que je n'arrive pas à mieux gérer le changement climatique (item 5).	.61			
Je me mets à l'écart et pense aux raisons pour lesquelles je me sens ainsi face au changement climatique (item6).	.64			
J'écris mes réflexions sur le changement climatique et je les analyse (item 7).	.39			
Je me demande pourquoi je réagis ainsi au changement climatique (item 8).	.58			
Mes préoccupations au sujet du changement climatique m'empêchent de m'amuser avec ma famille ou mes amis (item 9).		.78		
J'ai du mal à trouver un équilibre entre mes préoccupations pour le « durable » et les besoins de ma famille (item 10).		.53		
Mes préoccupations à propos du changement climatique interfèrent avec ma capacité à faire mon travail ou mes tâches scolaires (item 11).		.71		
Mes préoccupations concernant le changement climatique impactent négativement ma capacité à exploiter pleinement mon potentiel (item 12).		.82		
Mes amis disent que je pense trop au changement climatique (item 13).		.57		
J'ai été directement affecté par le changement climatique (item 14).			.89	
Je connais quelqu'un qui a été directement affecté par le réchauffement climatique (item 15).			.85	

J'ai constaté l'altération d'un lieu important pour moi suite au changement climatique (item 16).	.55	
J’aurais aimé me comporter d’une façon plus respectueuse de l’environnement (item 17).		.29
Je recycle (item 18).		.54
J'éteins les lumières (item 19).		.43
J’essaye de réduire mes comportements qui contribuent au changement climatique (item 20).		.60
Je me sens coupable si je gaspille de l’énergie (item 21).		.55
Je crois que je peux faire quelque chose pour aider à faire face au problème du réchauffement climatique (item 22).		.42

Note. CEI = cognitive-emotional impairments; FI = Functional impairments; EX= Personal experience of climate change; BE= Behavioral engagement.

Table S3. **Factor Loadings for the 2-Factor Model (Study 1)**

Items	CEI Factor	FI Factor
	.69	
Il m’est difficile de m’endormir lorsque je pense au changement climatique (item2).	.76	
Je fais des cauchemars à propos du changement climatique (item 3).	.69	
Je me retrouve à pleurer à cause du changement climatique (item 4).	.69	
Je me demande pourquoi est-ce que je n’arrive pas à mieux gérer le changement climatique (item 5).	.61	
Je me mets à l'écart et pense aux raisons pour lesquelles je me sens ainsi face au changement climatique (item6).	.64	
J'écris mes réflexions sur le changement climatique et je les analyse (item 7).	.39	
Je me demande pourquoi je réagis ainsi au changement climatique (item 8).	.57	
Mes préoccupations au sujet du changement climatique m'empêchent de m'amuser avec ma famille ou mes amis (item 9).		.78
J’ai du mal à trouver un équilibre entre mes préoccupations pour le « durable » et les besoins de ma famille (item 10).		.52
Mes préoccupations à propos du changement climatique interfèrent avec ma capacité à faire mon travail ou mes tâches scolaires (item 11).		.74
Mes préoccupations concernant le changement climatique impactent négativement ma capacité à exploiter pleinement mon potentiel (item 12).		.82
Mes amis disent que je pense trop au changement climatique (item 13).		.57

Note. CEI = cognitive-emotional impairments; FI = Functional impairments

***Table S4*. Factor Loadings for the 1-Factor Model (Study1)**

Items	CAS13
Il m'est difficile de me concentrer lorsque je pense au changement climatique (item 1)	.69
Il m'est difficile de m'endormir lorsque je pense au changement climatique (item2).	.73
Je fais des cauchemars à propos du changement climatique (item 3).	.68
Je me retrouve à pleurer à cause du changement climatique (item 4).	.68
Je me demande pourquoi est-ce que je n'arrive pas à mieux gérer le changement climatique (item 5).	.59
Je me mets à l'écart et pense aux raisons pour lesquelles je me sens ainsi face au changement climatique (item6).	.62
J'écris mes réflexions sur le changement climatique et je les analyse (item 7).	.40
Je me demande pourquoi je réagis ainsi au changement climatique (item 8).	.56
Mes préoccupations au sujet du changement climatique m'empêchent de m'amuser avec ma famille ou mes amis (item 9).	.74
J'ai du mal à trouver un équilibre entre mes préoccupations pour le « durable » et les besoins de ma famille (item 10).	.52
Mes préoccupations à propos du changement climatique interfèrent avec ma capacité à faire mon travail ou mes tâches scolaires (item 11).	.70
Mes préoccupations concernant le changement climatique impactent négativement ma capacité à exploiter pleinement mon potentiel (item 12).	.78
Mes amis disent que je pense trop au changement climatique (item 13).	.56

Table S5. **Comparison of three CFA Models without item 7 (Study 1)**

**Model**	Satorra Bentler χ^2	df	SRMR	RMSEA	RMSEA 90% CI	CFI	TLI	AIC	ECVI
4-Factor	373.72 **	183	.06	.06	.054–.072	.90	.88	15523.48	1.73
**2-Factor**	**122.82****	**53**	**.04**	**.07**	**.060–.095**	**.93**	**.92**	**8472.90**	**0.72**
1-Factor	160.69**	54	.05	.09	.078–.111	.90	.88	8520.69	0.87

Note. 4-Factor = four-factor model of the 22-item scale; 2-Factor = two-factor model of the 13-item scale; 1-Factor = single-factor model of the 13-item scale. df = degree of freedom; CI= confidence interval; SRMR = Standardized Root Mean Square Residual; RMSEA = Root Mean Square Error of Approximation; CFI = Comparative Fit Index; TLI = Tucker Lewis Index; AIC = Akaike Information Criterion; ECVI=Expected Cross-Validation Index. The best fitting model is shown in bold. ** $p < .01$.

Table S6. Comparison of three CFA Models without the outliers (Study1)

**Model**	Satorra Bentler X2	df	SRMR	RMSEA	RMSEA 90% CI	CFI	TLI	AIC	ECVI
4-Factor	416.96 **	203	.07	.06	.056–.073	.87	.86	15697.93	1.97
**2-Factor**	**139.32****	**64**	**.05**	**.07**	**.057–.090**	**.91**	**.89**	**8763.53**	**0.82**
1-Factor	173.30**	65	.06	.09	.072–.105	.88	.85	8806.06	0.96

Note. 4-Factor = four-factor model of the 22-item scale; 2-Factor = two-factor model of the 13-item scale; 1-Factor = single-factor model of the 13-item scale. df = degree of freedom; CI= confidence interval; SRMR = Standardized Root Mean Square Residual; RMSEA = Root Mean Square Error of Approximation; CFI = Comparative Fit Index; TLI = Tucker Lewis Index; AIC = Akaike Information Criterion; ECVI=Expected Cross-Validation Index. The best fitting model is shown in bold. ** $p < .01$.

***Table S7.* Descriptive Statistics for each item (Study 2)**

Items	Mean	SD	Min	Max	Range	Skewness	Kurtosis
CAS1	2.53	1.08	1	5	4	0.13	-0.79
CAS2	2.35	1.09	1	5	4	0.36	-0.69
CAS3	1.52	0.80	1	5	4	1.44	1.36
CAS4	1.59	0.89	1	5	4	1.34	0.85
CAS5	2.51	1.23	1	5	4	0.22	-1.13
CAS6	2.06	1.07	1	5	4	0.68	-0.53
CAS7	1.63	0.99	1	5	4	1.45	1.06
CAS8	1.96	1.07	1	5	4	0.81	-0.43
CAS9	2.21	1.07	1	5	4	0.45	-0.67
CAS10	2.86	1.17	1	5	4	-0.14	-0.93
CAS11	2.01	1.06	1	5	4	0.78	-0.25
CAS12	2.02	1.11	1	5	4	0.82	-0.28
CAS13	2.20	1.20	1	5	4	0.61	-0.75
CAS14	1.97	1.09	1	5	4	0.88	-0.14
CAS15	2.08	1.19	1	5	4	0.77	-0.50
CAS16	2.60	1.29	1	5	4	0.23	-1.09
CAS17	3.51	1.09	1	5	4	-0.45	-0.40
CAS18	4.54	0.70	1	5	4	-1.56	2.33
CAS19	4.61	0.65	1	5	4	-1.71	2.97
CAS20	4.31	0.77	1	5	4	-1.12	1.63
CAS21	3.94	1.06	1	5	4	-0.80	-0.15
CAS22	3.66	1.14	1	5	4	-0.47	-0.64

Table S8. **Factor Loadings for the 4-Factor Model (Study 2)**

Items	CEI Factor	FI Factor	EX Factor	BE Factor
Il m'est difficile de me concentrer lorsque je pense au changement climatique (item 1)	.69			
Il m'est difficile de m'endormir lorsque je pense au changement climatique (item2).	.69			
Je fais des cauchemars à propos du changement climatique (item 3).	.50			
Je me retrouve à pleurer à cause du changement climatique (item 4).	.54			
Je me demande pourquoi est-ce que je n'arrive pas à mieux gérer le changement climatique (item 5).	.59			
Je me mets à l'écart et pense aux raisons pour lesquelles je me sens ainsi face au changement climatique (item6).	.68			
J'écris mes réflexions sur le changement climatique et je les analyse (item 7).	.31			
Je me demande pourquoi je réagis ainsi au changement climatique (item 8).	.49			
Mes préoccupations au sujet du changement climatique m'empêchent de m'amuser avec ma famille ou mes amis (item 9).		.76		
J'ai du mal à trouver un équilibre entre mes préoccupations pour le « durable » et les besoins de ma famille (item 10).		.56		
Mes préoccupations à propos du changement climatique interfèrent avec ma capacité à faire mon travail ou mes tâches scolaires (item 11).		.77		
Mes préoccupations concernant le changement climatique impactent négativement ma capacité à exploiter pleinement mon potentiel (item 12).		.74		
Mes amis disent que je pense trop au changement climatique (item 13).		.60		
J'ai été directement affecté par le changement climatique (item 14).			.83	
Je connais quelqu'un qui a été directement affecté par le réchauffement climatique (item 15).			.85	

J'ai constaté l'altération d'un lieu important pour moi suite au changement climatique (item 16).			.50	
J’aurais aimé me comporter d’une façon plus respectueuse de l’environnement (item 17).				.30
Je recycle (item 18).				.49
J'éteins les lumières (item 19).				.43
J’essaye de réduire mes comportements qui contribuent au changement climatique (item 20).				.66
Je me sens coupable si je gaspille de l’énergie (item 21).				.65
Je crois que je peux faire quelque chose pour aider à faire face au problème du réchauffement climatique (item 22).				.51

Note. CEI = cognitive-emotional impairments; FI = Functional impairments; EX= Personal experience of climate change; BE= Behavioral Engagement.

***Table S9.* Factor Loadings for the 2-Factor Model (study 2)**

Items	CEI Factor	FI Factor
	.69	
Il m'est difficile de m'endormir lorsque je pense au changement climatique (item2).	.68	
Je fais des cauchemars à propos du changement climatique (item 3).	.49	
Je me retrouve à pleurer à cause du changement climatique (item 4).	.53	
Je me demande pourquoi est-ce que je n'arrive pas à mieux gérer le changement climatique (item 5).	.59	
Je me mets à l'écart et pense aux raisons pour lesquelles je me sens ainsi face au changement climatique (item6).	.68	
J'écris mes réflexions sur le changement climatique et je les analyse (item 7).	.31	
Je me demande pourquoi je réagis ainsi au changement climatique (item 8).	.50	
Mes préoccupations au sujet du changement climatique m'empêchent de m'amuser avec ma famille ou mes amis (item 9).		.76
J'ai du mal à trouver un équilibre entre mes préoccupations pour le « durable » et les besoins de ma famille (item 10).		.56
Mes préoccupations à propos du changement climatique interfèrent avec ma capacité à faire mon travail ou mes tâches scolaires (item 11).		.77
Mes préoccupations concernant le changement climatique impactent négativement ma capacité à exploiter pleinement mon potentiel (item 12).		.75
Mes amis disent que je pense trop au changement climatique (item 13).		.59

***Note*.** CEI = cognitive-emotional impairments; FI = Functional impairments

Table S10. **Factor Loadings for the 1-Factor Model (Study2)**

Items	CAS13
Il m'est difficile de me concentrer lorsque je pense au changement climatique (item 1)	.65
Il m'est difficile de m'endormir lorsque je pense au changement climatique (item2).	.62
Je fais des cauchemars à propos du changement climatique (item 3).	.44
Je me retrouve à pleurer à cause du changement climatique (item 4).	.48
Je me demande pourquoi est-ce que je n'arrive pas à mieux gérer le changement climatique (item 5).	.56
Je me mets à l'écart et pense aux raisons pour lesquelles je me sens ainsi face au changement climatique (item6).	.67
J'écris mes réflexions sur le changement climatique et je les analyse (item 7).	.32
Je me demande pourquoi je réagis ainsi au changement climatique (item 8).	.48
Mes préoccupations au sujet du changement climatique m'empêchent de m'amuser avec ma famille ou mes amis (item 9).	.72
J'ai du mal à trouver un équilibre entre mes préoccupations pour le « durable » et les besoins de ma famille (item 10).	.54
Mes préoccupations à propos du changement climatique interfèrent avec ma capacité à faire mon travail ou mes tâches scolaires (item 11).	.72
Mes préoccupations concernant le changement climatique impactent négativement ma capacité à exploiter pleinement mon potentiel (item 12).	.71
Mes amis disent que je pense trop au changement climatique (item 13).	.59

Table S11. **Comparison of three CFA Models without item 7 (Study 2)**

**Model**	Satorra Bentler χ^2	df	SRMR	RMSEA	RMSEA 90% CI	CFI	TLI	AIC	ECVI
4-Factor	896.56 **	183	.07	.06	.064–.074	.86	.84	49605.67	1.19
**2-Factor**	**345.62****	**53**	**.05**	**.87**	**.079–.096**	**.90**	**.88**	**28689.19**	**0.53**
1-Factor	502.69**	54	.06	.10	.099–.103	.85	.82	28884.04	0.75

Note. 4-Factor = four-factor model of the 22-item scale; 2-Factor = two-factor model of the 13-item scale; 1-Factor = single-factor model of the 13-item scale. df = degree of freedom; CI= confidence interval; SRMR = Standardized Root Mean Square Residual; RMSEA = Root Mean Square Error of Approximation; CFI = Comparative Fit Index; TLI = Tucker Lewis Index; AIC = Akaike Information Criterion; ECVI=Expected Cross-Validation Index. The best fitting model is shown in bold. $^{**}p < .01$.

***Table S12.* Comparison of three CFA Models without the outliers (Study2)**

**Model**	Satorra Bentler χ^2_2	df	SRMR	RMSEA	RMSEA 90% CI	CFI	TLI	AIC	ECVI
4-Factor	952.34 **	203	.07	.06	.063–.072	.86	.84	51724.77	1.28
**2-Factor**	**387.14****	**64**	**.05**	**.08**	**.076–.092**	**.90**	**.87**	**30851.04**	**0.60**
1-Factor	538.93**	65	.06	.10	.093–.109	.84	.80	31031.18	0.80

***Note*.** 4-Factor = four-factor model of the 22-item scale; 2-Factor = two-factor model of the 13-item scale; 1-Factor = single-factor model of the 13-item scale. df = degree of freedom; CI= confidence interval; SRMR = Standardized Root Mean Square Residual; RMSEA = Root Mean Square Error of Approximation; CFI = Comparative Fit Index; TLI = Tucker Lewis Index; AIC = Akaike Information Criterion; ECVI=Expected Cross-Validation Index. The best fitting model is shown in bold. **$p < .01$.

Table S13. **Correlations between the Climate Change Anxiety subscales (Study 2)**

	CEI (Factor 1)	FI **(**Factor 2)
CAS (13-item total)	.93**[.92–.94]	.89**[.87–.90]
CEI (Factor 1)		.66** [.63–.70]
FI (Factor 2)	.66**[.63–.70]	
PE (Factor 3)	.28**[.22–.34]	.27**[.21–.33]
BE (Factor 4)	.37**[.31–.42]	.33**[.27–.3]

Note. CEI = cognitive-emotional impairments; FI = Functional impairments; EX= Personal experience of climate change; BE= Behavioral Engagement; BDI = Beck depression inventory. GAD =general anxiety disorders; EID= environmental identity; CAS = climate anxiety scale. *$p < .05$ (corrected for multiple comparison using the Benjamini-Hochberg procedure).
